# Exploring social influences on the joint Simon task: empathy and friendship

**DOI:** 10.3389/fpsyg.2015.00962

**Published:** 2015-07-09

**Authors:** Ruth M. Ford, Bradley Aberdein

**Affiliations:** ^1^Department of Psychology, Anglia Ruskin UniversityCambridge, UK; ^2^School of Applied Psychology, Griffith University, BrisbaneQLD, Australia

**Keywords:** joint Simon effect, joint action, task co-representation, referential coding, perspective taking, empathy

## Abstract

Tasks for which people must act together to achieve a goal are a feature of daily life. The present study explored social influences on joint action using a Simon procedure for which participants (*n* = 44) were confronted with a series of images of hands and asked to respond via button press whenever the index finger wore a ring of a certain color (red or green) regardless of pointing direction (left or right). In an initial *joint condition* they performed the task while sitting next to another person (friend or stranger) who responded to the other color. In a subsequent *individual condition* they repeated the task on their own; additionally, they completed self-report tests of empathy. Consistent with past research, participants reacted more quickly when the finger pointed toward them rather than their co-actor (the *Simon Effect* or *SE*). The effect remained robust when the co-actor was no longer present and was unaffected by degree of acquaintance; however, its magnitude was correlated positively with empathy *only* among friends. For friends, the SE was predicted by cognitive perspective taking when the co-actor was present and by propensity for fantasizing when the co-actor was absent. We discuss these findings in relation to social accounts (e.g., task co-representation) and non-social accounts (e.g., referential coding) of joint action.

## Introduction

Social activities often require careful co-ordination of behaviors between people, for example, when they dance together, play games or competitive sport, work in unison to build things, and engage in cultural transmission of artifacts and technology. Given the ubiquity of such activities in daily life, understanding the mechanisms of effective collaboration is essential. In laboratory studies, one of the most widely used paradigms for investigating joint action has been the joint Simon task devised by Sebanz et al. (2003).

In an individual Simon task, participants are asked to respond to the color of stimuli presented on a computer monitor (e.g., pressing a left key when a red stimulus appears or pressing a right key when a green stimulus appears) while ignoring the spatial location of the stimulus (left versus right of the monitor). The term *Simon Effect (SE)* refers to the finding that participants react faster when the spatial relationship between stimulus and response is compatible (e.g., pressing a left key in response to a stimulus on the left) than when it is incompatible (e.g., pressing a left key in response to a stimulus on the right; review by Lu and Proctor, 1995). Although the SE typically vanishes when participants are asked to respond to only one stimulus color in a go/no-go version of the task, it reappears during the joint Simon task – that is, when they perform their role while seated next to another person who is responsible for carrying out the alternative response (e.g., with the person seated on the left responding solely to red stimuli and the person sitting on the right responding solely to green stimuli). The phenomenon of a spatial compatibility effect under joint-action conditions has been termed the *joint SE* (review by Dolk et al., 2014).

Sebanz et al. (2003, 2005a) speculated that the joint SE arises because participants internalize their partner’s contribution to the activity, thus generating a shared task representation that interferes with their responses to ‘go’ signals when the irrelevant stimulus dimension primes the co-actor’s role instead. This suggestion is in line with *ideomotor theory*, which supposes that action and action perception are closely coupled and, hence, that either observing or anticipating another person’s motor responses activates the same representational structures as if the responses were self-generated (e.g., Jeannerod, 1999). A related account holds that the joint SE reflects *actor* co-representation rather than *task* co-representation; specifically, difficulties are attributed to the fact that participants have to decide whose turn is signaled on each trial rather than because they mentally depict exactly what their co-actor is supposed to do (Philipp and Prinz, 2010; Wenke et al., 2011).

Sebanz et al. (2003) discovered that there is no need for on-line feedback about the co-actor’s responses to produce the joint SE. Because the effect was robust when the co-actor performed the complementary role but their hand was hidden from view, results were taken to mean that the joint SE is triggered when participants first encode their co-actor’s contribution to the task. It has further been shown that the joint SE appears even when participants sit outside each other’s peripersonal space (Welsh et al., 2013). However, there is conflicting evidence regarding whether participants need to be able to see each other at all. The joint SE has been observed when participants perform the task from different rooms (Tsai et al., 2008), and when they are prevented from seeing and hearing each other during the activity (Vlainic et al., 2010). In contrast, two studies found no joint SE when co-actors were physically separated (Welsh et al., 2007; Sellaro et al., 2013).

Notably, the size of the joint SE has been shown to vary according to the interpersonal relationship of the co-actors. For example, it is accentuated when participants are paired with a partner who is friendly and supportive rather than unfriendly and intimidating (Hommel et al., 2009), or who is perceived as an in-group member rather than out-group member (Müller et al., 2011b; McClung et al., 2013). It is similarly increased when participants are engaged in an activity that encourages thoughts of inter-personal connectedness (Colzato et al., 2012a), promotes a positive- rather than negative mood (Kuhbandner et al., 2010), or encourages interdependence rather than autonomy (Ruys and Aarts, 2010). It has even been reported that Buddhists showed a greater joint SE than atheists, a phenomenon attributed to heightened awareness of, and caring for, other people in the former group (Colzato et al., 2012b). Although the joint SE fails to emerge when participants perform the task together with a mechanical agent (Tsai et al., 2008), it can be restored if the agent is described as “active and intelligent” rather than “passive and purely deterministic” (Stenzel et al., 2012) or is endowed with other human characteristics (Müller et al., 2011a).

Several studies have evaluated the joint SE in participants who have profound impairments of social cognition, such as individuals with autism spectrum disorder (ASD), schizophrenia, or brain injury. In a study that tested high-functioning adults with ASD, Sebanz et al. (2005b) noted that the effect was intact – a finding they interpreted to mean that individuals with ASD have normal capacity for mapping observed actions onto mental representations of self-performed actions. In contrast, the joint SE failed to emerge in patients with schizophrenia (Liepelt et al., 2012) whereas mixed findings were obtained with brain-damaged adults who performed poorly on theory-of-mind tests (Humphreys and Bedford, 2011). The latter study found that participants did not show a joint SE given standard task instructions although the effect emerged (to some extent) if they were requested to pay particular attention to their co-actor. Based on these results, Humphreys and Bedford (2011) concluded that the joint SE is linked to social-cognitive abilities. They further speculated that the reason Sebanz et al. (2005b) *did* find a joint SE in their ASD sample was because these high-functioning participants generally succeeded in passing tests of first- and second-order belief attribution.

The evidence reviewed so far implicates an important contribution of social factors to the joint SE, consistent with notions about task and agent co-representation. Nevertheless, there are grounds for querying whether it is truly a social phenomenon. Guagnano et al. (2010) suggested that the joint SE merely reflects participants’ spatial coding of the activity, coding that is heightened when the experimental conditions emphasize collaboration rather than individual performance. On their account, participants do not consider their co-actor’s agency or intentionality when performing the joint Simon task; rather, the joint SE emerges because the co-actor provides a frame of reference that transforms the participant’s perception of their role from *simple* button press to either *left or right* button press (see also, Dittrich et al., 2012, 2013). Consistent with this interpretation, Guagnano et al. (2010) showed that joint SE depends on the physical proximity of the co-actors, with greater separation associated with a smaller effect.

Alternatively, a *referential coding* argument draws on the theory of event coding (TEC: Hommel et al., 2001) in proposing that the joint SE reflects conflict between concurrently active event representations (which comprise information regarding the perceptual characteristics of the actions and their sensory consequences). This concurrent activation is thought to pose a discrimination problem that participants attempt to solve by focusing on features that best differentiate the competing representations – which in a typical joint Simon task is usually the left/right location of the responses (Dolk et al., 2011, 2014). Unlike the spatial response coding explanation, a referential coding view succeeds in accounting for evidence that the social similarity of the two co-actors influences the magnitude of the joint SE; specifically, it is assumed that greater perceptual and conceptual overlap between event representations increases the likelihood that participants will try to distinguish between them by linking them with their respective spatial locations. In line with notions about referential coding, Dolk et al. (2013) obtained reliable SEs in an auditory version of the joint Simon procedure which emphasized the horizontal dimension using a variety of non-animate objects, including a Japanese waving cat, a clock, and a ticking metronome. The effect disappeared using an object that was less attention-grabbing (a silent metronome), ruling out the possibility that the placement of the objects by a human experimenter leant them social significance.

### The Present Study

In the present study we took a novel approach to evaluating social influences on the jointly performed Simon task by assessing participants for trait empathy. Despite evidence that the joint SE depends on participants’ mood (Kuhbandner et al., 2010) and relationship with the co-actor (Hommel et al., 2009; Müller et al., 2011b; McClung et al., 2013) no previous research has explored sources of individual differences in the size of the effect. Based on the aforementioned evidence, we expected that the magnitude of the joint SE would be greater among participants who are more empathic.

We evaluated empathy comprehensively by measuring both cognitive and affective components. Whereas *cognitive empathy* involves the ability to ponder other people’s intentions and beliefs (i.e., theory-of-mind), *affective empathy* reflects our immediate emotional responsiveness to others’ feelings. These two types of empathy appear to rely on different brain systems; neuroimaging evidence suggests that cognitive empathy engages the temporo-parietal junction, medial parietal cortex, and medial prefrontal cortex, with affective empathy instead relying on the anterior midcingulate cortex, dorsal anterior cingulate cortex, and bilateral anterior insula (Frith and Frith, 2003; Bernhardt and Singer, 2012) and possibly involving a mirror neuron system that elicits spontaneous mimicry of others’ observed emotional states (Iacoboni, 2009). In relation to the Simon task, previous research suggests that cognitive empathy is likely to have a greater influence on the joint SE. An impact of cognitive empathy is implicated by the finding that the effect is lacking among brain-injured patients who fail theory-of-mind tests (Humphreys and Bedford, 2011). Moreover, the theory-of-mind network handles the reading of others’ intentions and appears to play an important role in maintaining the distinction between self and other (review by Decety and Sommerville, 2003).

Importantly, we also explored whether any possible influence of empathy on the joint SE was affected by the degree of prior acquaintance between the co-actors; specifically, we were interested in the possibility that effects of empathy might be *accentuated for actors who were well acquainted*. Given evidence that the perception of self-other overlap is increased when people know each other well (e.g., Myers and Hodges, 2012), we asked participants to perform the joint Simon task with either a friend (who signed up for the experiment at the same time) or a stranger (who signed up individually). The need to take account of relational context when researching human social behavior has been gaining recognition, especially in regards to kinship and friendship (reviews by Beckes and Coan, 2013; Clark-Polner and Clark, 2014). People empathize more readily with those who are familiar to them or who share similar social characteristics (Preston and de Waal, 2002; Decety and Lamm, 2006). For example, it has been reported that brain activations in response to self-focused threat mimic those elicited by threat to a friend but not by threat to a stranger (Beckes et al., 2013) and that the physiological arousal experienced by fire-walkers is tightly bound with that of the spectators *only* when such spectators are friends or relatives (Konvalinka et al., 2011). Such evidence indicates that the perception/action coupling mechanisms underpinning empathy are modulated by brain centers involved in processing of social information and, consequently, that the experience of empathy is heightened when the neural representations of self- versus other show greater overlap (Beckes et al., 2013). It therefore follows that *if* empathy influences the joint SE *then* the trend should be augmented when the co-actors are friends.

After completing the joint version of the Simon task, all participants were asked to perform the same task individually; that is, seated in the same chair as before and making the same response they had been responsible for in the earlier joint version. Although previous studies have observed no spatial compatibility effect in the individual (one-handed, go/no-go) Simon task, we wanted to see whether this would still hold true if the individual condition followed the joint condition and conformed to its procedure in all respects apart from the absence of the co-actor. Both social and non-social accounts of the joint SE suggest that the effect is likely to endure under these conditions. On a social account, such a scenario could give rise to thoughts of the co-actor performing their complementary role that reinforce participants’ task sharing or agent co-representation. On a non-social account, by contrast, the participants could carry forward with them the spatial response mappings that they acquired during the earlier joint procedure – a possibility in line with evidence that the SE emerges in the individual go/no-go Simon task when participants have recently performed the standard two-handed version (Ansorge and Wühr, 2009). In such an eventuality it will be of interest to compare the relations between empathy and the SE in the joint and individual conditions. For example, if empathy predicts the magnitude of the effect *only* when participants perform the task in the company of their co-actor then this could suggest that the joint task elicits genuine social processes that are lacking in the individual task.

## Materials and Methods

### Participants

Participants were 44 undergraduate students at Griffith University, ranging in age from 17- to 44 years (*M* = 22.50, SD = 5.49), who received course credit or a small payment depending on how they were recruited. All participants had normal or corrected-to-normal near vision, were not red–green color blind, and had no impairments of manual co-ordination. They either signed up as part of a friendship pair (*n* = 22; 15 females; mean age = 21.5) or were assigned to the stranger condition (*n* = 22; 17 females; mean age = 23.5), with 11 pairs (10 same-gender and one mixed-gender) in each case. The ‘friend’ and ‘stranger’ groups did not differ significantly in terms of either age, *t*(42) = -1.27, *p* = 0.210, or gender ratio, χ^2^(1, *N* = 22) = 0.46, *p* = 0.498.

### Materials and procedure

Procedures for the study were approved by the Griffith University Human Research Ethics Committee. Upon arrival at the laboratory, participants who had signed up for the stranger condition were introduced to their partner for the joint task. Otherwise, the researcher questioned the participants who had signed up for the friend condition to ensure that they met the criteria for inclusion in this condition (i.e., close friends who socialized with one another often and had known each other for at least 6 months). Following this process, participants completed four activities in a single test session lasting no more than an hour. These comprised (1) the joint Simon task, (2) the individual Simon task, (3) the Empathy Quotient (EQ), and (4) the Interpersonal Reactivity Index (IRI). All participants completed the joint version of the Simon task as the first activity of the test session. Following this, one member of each pair performed the individual version while the other completed the empathy questionnaires; subsequently, their roles were reversed. For participants who performed the individual task straight after the joint task, there was a break of 3–4 min while the researcher accompanied the co-actor to the adjacent room and explained the procedure for completing the empathy questionnaires. For the remaining participants, the individual task was performed around 15 min after the joint task (the average time taken to complete the empathy questionnaires). On each occasion the Simon task comprised a block of eight practice trials followed by 200 test trials.

#### Joint and Individual Simon Tasks

**FIGURE 1:**
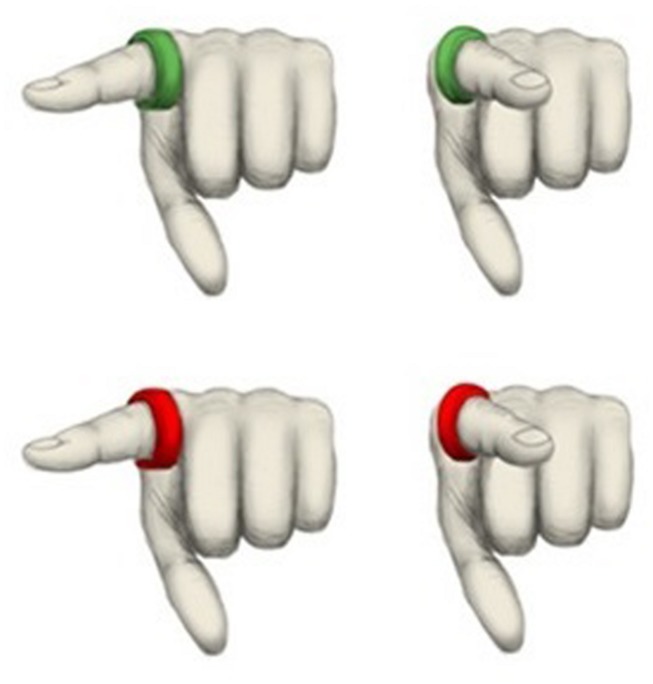
**Experimental stimuli; Green pointing left **(top left)**, green pointing right **(top right)**, red pointing left **(bottom left)** and red pointing right (bottom right)**.

The joint and individual Simon tasks were administered using a Dell laptop computer with Windows XP operating system. The computer had a screen resolution of 1440 × 1020 pixels; the program was run at this same screen resolution. A pair of Microsoft branded computer mice connected to the laptop via the audio and microphone jacks were used as the input devices for responding to the stimuli. These computer mice were programmed to enable simultaneous input, to the same stimulus, into the computer. Participants viewed a series of hands presented on the computer monitor that varied in terms of ring color (green versus red) and pointing direction of the index finger (left versus right). This factorial design yielded four different images, namely (1) green ring/pointing left, (2) green ring/pointing right, (3) red ring/pointing left and (4) red ring/pointing right (see **Figure 1**). Participants were requested to press the button on their mouse *either* in response to a red ring (22 participants) *or* in response to a green ring (22 participants), regardless of pointing direction. In the former case, for example, red rings signal ‘go’ trials and green rings signal ‘no go’ trials. Following the eight practice trials, participants saw 200 images in total (50 each of four stimulus types) that were presented in random order, with the proviso that the same color ring could not appear more than three times in succession.

For the joint condition, participants were seated next to each other (with a separation of around 30 cm) in front of the computer monitor with their preferred index finger resting on the left button of their mouse. Initially, a set of instructions appeared on the screen that described the procedure as follows, “You will see a series of hands appear on the monitor one at a time. Each hand will be pointing either to the left or to the right and will be wearing either a green ring or a red ring. The person sitting on the left should press their button as quickly as possible whenever the hand is wearing a GREEN ring. The person sitting on the right should press their button as quickly as possible whenever the hand is wearing a RED ring. Your reaction times (RTs) and accuracy will be recorded. Please place the index finger of your dominant hand on your button and get ready to start.” The capitalized color words were either green or red as appropriate whereas the remaining text was black. After practicing the task, participants were given the opportunity to ask questions to clarify aspects of the procedure before the researcher initiated the test phase. For all participant pairs, the person seated on the left responded to the green ring whereas the person seated on the right responded to the red ring.

For the subsequent individual condition, participants were requested to respond to the same color ring as for the joint condition. Also, they remained in the same seat on the same side of the monitor as previously. Thus, the individual condition differed from the joint condition *only* in that the participant completed the task while sitting next to the now-vacated chair of their partner. Instructions mimicked those provided for the joint condition except that only one participant was mentioned. For example, instructions for individuals assigned to the green ring condition were as follows, “You will see a series of hands appear on the monitor one at a time. Each hand will be pointing either to the left or to the right and will be wearing either a green ring or a red ring. Your task is to press the button as quickly as possible whenever the hand is wearing a GREEN ring.”

The sequence of events on each individual trial of the task was as follows: (1) a fixation point (a black cross) displayed for 100 ms, (2) a blank (white) screen displayed for 100ms, (3) the target stimulus displayed for 500 ms, and (4) a further blank screen displayed for 500 ms. Recording of the RTs was initiated from the instant that the stimulus appeared, giving participants 1000 ms to respond before the next trial commenced. Compatible trials were those in which the finger was aimed at the player receiving a ‘go’ signal based on ring color (i.e., a finger pointing left when wearing a green ring, a finger pointing right when wearing a red ring) and incompatible trials were those in which the finger was aimed at the player receiving a ‘no go’ signal based on ring color (i.e., a finger pointing right when wearing a green ring, a finger pointing left when wearing a red ring).

#### Empathy Measures

Participants completed two empathy questionnaires in an adjacent room while their co-actor carried out the individual version of the Simon task.

##### Empathy quotient

The empathy quotient (EQ) is a self-report measure designed to assess empathy is normal adult populations (Baron-Cohen and Wheelwright, 2004). It comprises 60 rating-scale questions (40 target questions intermixed with 20 distracter questions) that yield an overall score encompassing cognitive perspective taking, affective empathy, and social skills.

##### Interpersonal reactivity index

The IRI is a self-report measure of cognitive and affective empathy using 28 rating-scale questions that yield four separate scores; empathetic concern (i.e., the ability to feel concern for others), perspective taking (i.e., the ability to understand another person’s point of view), fantasy (i.e., one’s propensity for becoming involved in fiction and fictional characters) and personal distress (i.e., feelings that accompany altruistic behaviors; Davis, 1980). Perspective taking and fantasy represent cognitive aspects of empathy whereas empathic concern and personal distress represent affective aspects of empathy.

## Results

Data were screened for normality and univariate outliers both across- and within groups (Tabachnick and Fidell, 2007). All empathy measures and Simon task variables used in the following analyses had distributions that were acceptably normal. Adopting a criterion of absolute *z* > 2.5, a single outlier was identified in the friends group; namely, an unusually low score on IRI Perspective Taking (*z* = -2.61). In the strangers group, there was one high outlier in terms of size of the joint SE (*z* = 3.19). Analyses were conducted both with and without these cases; because the pattern of findings was unaffected the analyses reported below included all participants’ data.

### Joint and Individual Simon Tasks

#### Reaction Times on ‘Go’ Trials

All participants responded accurately on all ‘go’ trials. **Table 1** shows group means and standard deviations of RTs in milliseconds, presented separately for compatible and incompatible trials in the joint condition (top panel) and the subsequent individual condition (bottom panel). The far-right columns show descriptive statistics for the SE, calculated by subtracting RTs on compatible trials from RTs on incompatible trials. Data were subjected to a 2 (Group: friends vs. strangers) × 2 (Trial Type: compatible vs. incompatible) × 2 (Player Condition: joint vs. individual) ANOVA with repeated measures on the second and third factors. There was a significant main effect for trial type, *F*(1,42) = 11.14, *p* = 0.002, $\eta_{p}^{2}$ = 0.21, reflecting faster response latencies on compatible trials (compatible *M* = 432.53, incompatible *M* = 438.07). In contrast, there were no significant effects of either group (friends *M* = 433.33, strangers *M* = 437.27), *F*(1,42) = 0.52, *p* = 0.474, $\eta_{p}^{2}$ = 0.01, or player condition (joint *M* = 435.34, individual *M* = 435.26), *F*(1,42) = 0.00, *p* = 0.980, $\eta_{p}^{2}$ = 0.00. Likewise, there were no reliable interactions, all *p*-values >0.05 (lowest *p* = 0.33).

**Table 1:** Group mean and SD of response latencies (in milliseconds) shown separately for compatible and incompatible trials in the joint condition (top panel) and the subsequent individual condition (bottom panel).

	Compatible		Incompatible		SE Difference Scores		
	*M*	SD	*M*	SD	*M*	SD	Range
**Joint Condition**		
Friends	429.68	14.42	438.59	22.64	8.91	19.66	-33 to 48
Strangers	435.05	20.53	438.05	23.53	3.00	13.32	-23 to 22
Overall	432.36	17.74	438.32	22.82	5.95	16.86	-33 to 48
**Individual Condition**		
Friends	429.82	25.93	435.23	24.83	5.41	14.09	-24 to 26
Strangers	435.59	24.34	440.41	20.75	4.82	13.08	-12 to 48
Overall	432.70	25.03	437.82	22.76	5.11	13.44	-24 to 48

#### False Alarms on ‘No Go’ Trials

Participants rarely responded inappropriately on ‘no go’ trials. There was no evidence that the frequency of such errors was affected by spatial compatibility (compatible trials *M* = 0.86, incompatible trials *M* = 0.91), *t*(43) = 0.36, *p* = 0.722, $\eta_{p}^{2}$ = 0.00, or player condition (joint *M* = 0.88, individual *M* = 0.90), *t*(43) = 0.17, *p* = 0.870, $\eta_{p}^{2}$ = 0.00. However, there were reliably fewer false alarms among friends (friends *M* = 0.65, strangers *M* = 1.13), *t*(42) = 2.50, *p* = 0.016, $\eta_{p}^{2}$ = 0.13.

#### Descriptive Statistics for the EQ and IRI

**Table 2** shows descriptive statistics of scores on the EQ and the four scales of the IRI. There were no significant group differences on any of the measures, all *p*-values >0.05 (lowest *p* = 0.18). Results for the whole sample closely approximate normative data reported in previous research (IRI: Davis, 1980; EQ: Lawrence et al., 2004). Outcomes for the EQ were positively correlated with those for IRI Perspective Taking although the effect was reliable only for strangers; friends *r*(22) = 0.40, *p* = 0.067; strangers *r*(22) = 0.51, *p* = 0.016. Similarly, the EQ was positively correlated with IRI Empathic Concern; friends *r*(22) = 0.44, *p* = 0.040; strangers *r*(22) = 0.72, *p* < 0.001. In contrast, the EQ was not significantly correlated with IRI Fantasy; friends *r*(22) = 0.06, *p* = 0.782; strangers *r*(22) = 0.21, *p* = 0.353, or IRI Personal Distress; friends *r*(22) = 0.27, *p* = 0.234; strangers *r*(22) = -0.19, *p* = 0.402. Likewise, Lawrence et al. (2004) found reliable correlations between the EQ and the IRI for the perspective taking scale and the empathic concern scale but not for the fantasy scale and the personal distress scale.

**Table 2:** Descriptive statistics of scores on the Empathy Quotient (EQ) and the four scales of the IRI.

	Friends			Strangers			Whole group		
	*M*	SD	Range	*M*	SD	Range	*M*	*SD*	Range
Full-Scale EQ	44.50	7.64	30–56	47.23	9.46	27–62	45.86	8.61	27–62
IRI: Perspective Taking	16.18	4.79	4–25	17.18	5.00	5–24	16.68	4.87	4–25
IRI: Fantasy Scale	18.05	5.32	5–24	16.59	5.76	5–27	17.32	5.53	5–27
IRI: Empathic Concern	20.23	3.99	9–28	20.23	4.59	10–28	20.23	4.25	9–28
IRI: Personal Distress	10.36	4.86	1–19	12.91	4.51	4–20	11.64	4.81	1–20

*Maximum possible scores are as follows: EQ = 80; Each IRI scale = 28.*

#### Exploring the SE as a Function of Empathy

**Table 3** shows Pearson correlations between (1) scores on the EQ and the four scales of the IRI, and (2) the magnitude of the SE in the joint- and individual conditions (calculated in each case by subtracting mean RT on compatible trials from mean RT on incompatible trials). The correlations are presented for the whole sample, as well as for friends and strangers separately (*n* = 22 each) to evaluate our prediction that any influence of empathy would be accentuated for friends.

**Table 3:** Bivariate correlations between (1) the magnitude of the SE, and (2) scores on the EQ and four scales of the IRI.

	EQ	IRI:PT	IRI:FS	IRI:EC	IRI:PD
**Whole group (*n* = 44)**		
Joint condition	0.23	0.36*	0.01	0.06	0.11
Individual condition	0.15	0.12	0.07	0.16	-0.13
**Friends (*n* = 22)**					
Joint condition	0.51*	0.56**	0.16	0.24	0.24
Individual condition	0.00	0.06	0.52*	0.14	0.20
**Strangers (*n* = 22)**		
Joint condition	0.00	0.17	-0.25	-0.17	0.04
Individual condition	0.30	0.18	-0.38	0.17	-0.50*

EQ, empathy quotient; IRI:PT, perspective taking; IRI:FS, fantasy scale; IRI:EC, empathic concern; IRI:PD, personal distress.

*p < 0.05 two-tail, **p < 0.01 two-tail; significant correlations are shown in bold.

When considering the SE for the joint condition, results for the whole sample showed that the effect was significantly and positively correlated with perspective taking as gaged by the IRI, *p* = 0.016. As predicted, however, the impact of empathy was enhanced among friends. For the friend pairs, the SE for the joint condition was correlated with *both* IRI Perspective Taking, *p* = 0.007, and scores on the EQ, *p* = 0.015. For the stranger pairs, by contrast, it failed to show a reliable correlation with any of the empathy measures.

When considering the SE for the individual condition, results for the whole sample were not correlated with empathy. Nevertheless, for friends the effect was augmented among participants who scored higher on IRI Fantasy, *p* = 0.014. For strangers, the only significant result was a *negative* correlation between the SE and IRI Personal Distress (i.e., participants who reported higher levels of personal distress were less likely to show a speed advantage on compatible trials relative to incompatible trials when performing the task on their own).

**FIGURE 2:**
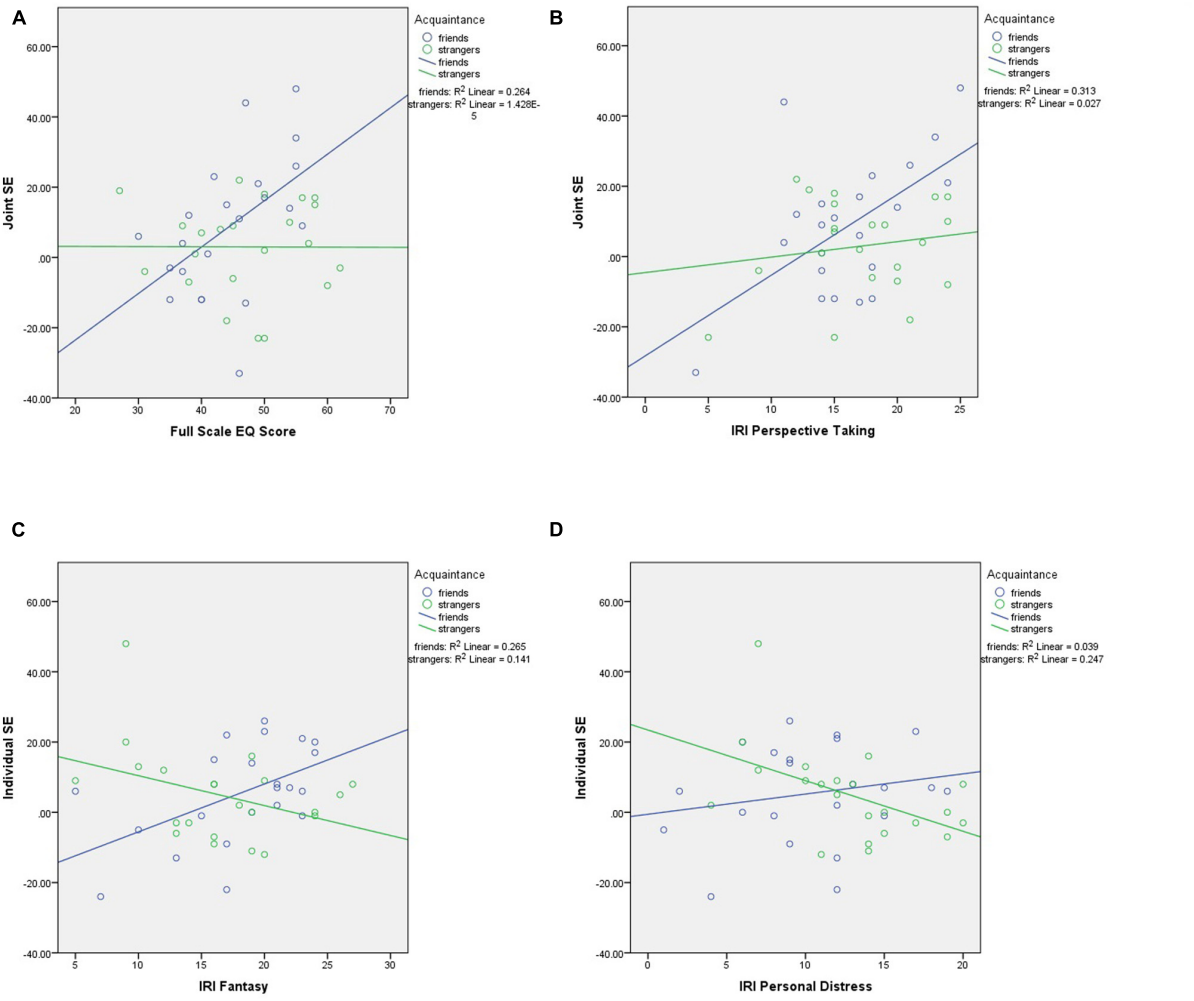
**Scatterplots showing the group × empathy interactions for **(A)** EQ and the joint condition Simon effect (SE), **(B)** perspective taking and the joint condition SE, **(C)** fantasizing and the individual condition SE, and **(D)** personal distress and the individual condition SE**.

To compare the impact of empathy on the SE between the two groups, ANCOVAs were conducted that entered SE as the dependent variable, group (i.e., friends or strangers) as the independent variable, and empathy measure as the covariate. These analyses were conducted for all empathy measures and for both the joint and individual SEs, and produced significant outcomes in the following four cases. First, the ANCOVA exploring group differences in the effects of EQ scores on the joint SE showed a marginal effect of group, *F*(1,40) = 4.03, *p* = 0.052, $\eta_{p}^{2}$ = 0.09, a significant effect of the EQ, *F*(1,40) = 5.30, *p* = 0.027, $\eta_{p}^{2}$ = 0.12, and a significant interaction, *F*(1,40) = 5.39, *p* = 0.025, $\eta_{p}^{2}$ = 0.12. Second, the ANCOVA exploring group differences in the effects of IRI Perspective Taking on the joint SE showed a non-significant effect of group, *F*(1,40) = 2.04, *p* = 0.161, $\eta_{p}^{2}$ = 0.05, a significant effect of IRI Perspective Taking, *F*(1,40) = 8.17, *p* = 0.007, $\eta_{p}^{2}$ = 0.17, and a marginal interaction, *F*(1,40) = 3.76, *p* = 0.059, $\eta_{p}^{2}$ = 0.09. Third, the ANCOVA exploring group differences in the effects of IRI Fantasy on the individual SE showed a significant effect of group, *F*(1,40) = 9.14, *p* = 0.004, $\eta_{p}^{2}$ = 0.19, a non-significant effect of IRI Fantasy, *F*(1,40) = 0.53, *p* = 0.465, $\eta_{p}^{2}$ = 0.01, and a significant interaction, *F*(1,40) = 10.22, *p* = 0.003, $\eta_{p}^{2}$ = 0.20. Fourth, the ANCOVA exploring group differences in the effects of IRI Personal Distress on the individual SE showed a significant effect of group, *F*(1,40) = 4.91, *p* = 0.033, $\eta_{p}^{2}$ = 0.11, a non-significant effect of IRI Personal Distress, *F*(1,40) = 1.02, *p* = 0.318, $\eta_{p}^{2}$ = 0.03, and a significant interaction, *F*(1,40) = 5.56, *p* = 0.023, $\eta_{p}^{2}$ = 0.12. **Figure 2** depicts the group × empathy interactions for (1) EQ and the joint SE, (2) IRI Perspective Taking and the joint SE, (3) IRI Fantasy and the individual SE, and (4) IRI Personal Distress and the individual SE.

## Discussion

Consistent with previous investigations (e.g., Sebanz et al., 2003), results for the joint condition showed a robust SE. Although participants merely had to respond to a particular color ring and withhold responding to the alternative color, they reacted more slowly when the finger wearing the ring pointed toward their co-actor than when it pointed in their own direction. This spatial compatibility effect emerged regardless of whether the co-actors were friends or strangers and remained reliable when participants were later asked to perform the task on their own.

But despite no effect of prior acquaintance on the strength of the joint SE, results of the present study were striking in showing that empathy influenced the performance of friends to a greater extent than strangers. When considering the whole sample, there was indeed a significant correlation between the joint SE and cognitive perspective taking as gaged by the IRI. However, this effect was moderated by group such that its magnitude was greater among friends than among strangers. Similarly, a significant interaction between group and the EQ reflected an augmented influence of general empathy on the joint SE in the case of friends. Group differences in the pattern of correlations could not be attributed to discrepant outcomes for empathy because friends and strangers performed equivalently on all empathy measures. For friends, the finding that the joint condition SE was predicted by the IRI perspective taking scale (indexing the extent to which responders strive to understand others’ thoughts, beliefs, and intentions) lends weight to observations of a reduced effect in clinical groups that characteristically perform poorly on theory-of-mind tests, such as patients with schizophrenia (Liepelt et al., 2012) or brain injury (Humphreys and Bedford, 2011).

It was further shown that the impact of empathy on the SE differed between friends and strangers even in the subsequent individual condition. When friends performed the task on their own, a larger SE was associated with higher scores on the fantasizing scale of the IRI – this effect was lacking in the strangers group as reflected in a significant interaction between group and fantasizing. In contrast, strangers showed a *negative* correlation between the individual SE and IRI Personal Distress that failed to emerge among friends.

Previous research has produced evidence of top–down modulation of empathy from brain centers that process information about social relationships (e.g., Preston and de Waal, 2002; Beckes et al., 2013; Decety, 2015). There is also a growing literature documenting effects of relational context on social behavior and neural responses (reviews by Beckes and Coan, 2013; Clark-Polner and Clark, 2014). In an electrophysiological study, Leng and Zhou (2010) measured event-related brain potentials while participants observed reward feedback to either a friend or a stranger in a gambling task. They found that a late component, the P300, was modulated by the acquaintance variable – suggesting that outcome evaluation engages a controlled process of cognitive appraisal that is sensitive to interpersonal relationships. Using functional magnetic resonance imaging (fRMI) during a social interaction simulation task, Güroğlu et al. (2008) reported that when participants interacted with friends rather than strangers they showed stronger activations in brain regions integral to empathy and reward-related processes. These findings highlight the effect of social bonds on the affective experience associated with everyday human interactions. More generally, they resonate with extensive developmental research documenting the important role of emotional attachments in stimulating joint attention and shared intentionality (Tomasello et al., 2005).

One possible conclusion to be drawn from our study is that the underlying mechanisms of the joint SE differed between friends and strangers, with the former group showing a greater influence of processes that were genuinely social. Assuming this to be true, it follows that conclusions about joint action might differ profoundly depending on how well the co-actors know one another. It has been suggested that the basic human drive to form friendships might have originated in the evolutionary advantage afforded to cooperative- over individual activities (Baumeister and Leary, 1995). From this perspective, social relationships and joint action could be linked in a mutually reinforcing manner, in keeping with evidence that encouraging participants to engage in synchronized movement promotes emotional closeness and improves their ability to pursue joint action goals (Valdesolo et al., 2010).

On a social account, members of the friends group who had better perspective-taking skills might have been more inclined to note their co-actor’s role in the task – thus increasing their susceptibility to interference when the pointing direction of index finger signified the co-actor’s response (consistent with notions of task co-representation; Sebanz et al., 2005a). Alternatively, their proclivity for putting themselves in others’ shoes could have fostered a sense of collaboration with the co-actor that posed challenges of agent identification on incompatible trials (as suggested by notions of agent co-representation; Philipp and Prinz, 2010; Wenke et al., 2011). Consistent with the latter interpretation, Wenke et al. (2011) obtained preliminary evidence linking general empathy with impairments of self/other differentiation. In their study, participants (who faced one another) were each responsible for binary go/no-go responses to a central colored circle (the target) flanked by two other circles. For example, one participant responded to a blue target with a left key press and to a red target with a right key press, whereas the other participant responded to a yellow target with a left key press and to a green target with a right key press. Although irrelevant to the participant’s response, the flanker circles could be either ‘own color’ or ‘other color’ and signal either the same key press (compatible trial) or the opposing key press (incompatible trial). Whereas results failed to support the hypothesis that participants would be influenced by knowledge of their co-actor’s specific stimulus/response mappings, the finding that RTs on ‘go’ trials were faster when targets appeared next to ‘own’ flankers rather than ‘other’ flankers was taken to mean that the flankers influenced the ease of agent identification. Because the own-flanker advantage was accentuated for participants who scored highly on self-reported empathy, Wenke et al. (2011) further posited that agent identification was impaired by empathic processes.

On the other hand, it could be argued that the joint SE was driven by referential coding in both groups but that event representations differed between friends and strangers. From the perspective of the referential encoding account, action control during joint tasks poses a discrimination problem; specifically, participants are thought to represent events they produce themselves in the same manner as events that are not under their control regardless of whether the external events are of human origin. It is assumed that event representations comprise information about the characteristics and consequences of actions, and that action selection involves activating codes of to-be-generated action effects (Hommel et al., 2001). The problem of selecting the relevant representation from all currently active representations is made more difficult when the sources are perceptually or conceptually similar, thus heightening participants’ attention to whichever spatial features of the task help to differentiate between self- and other-generated events (Dolk et al., 2011, 2014). Applied to the present findings, notions about referential coding raise the possibility that friends assigned greater prominence than strangers to features within their event representations that were socially relevant; consequently, friends who were more empathic may have perceived greater similarity between self- and other-generated events than did friends who were less empathic. In the case of strangers, by contrast, overlap between competing event representations may have reflected different kinds of attributes (perhaps more perceptual than conceptual) that were not indexed by any of our individual differences measures.

As expected, there were no indications that the joint SE for friends was influenced by *affective* empathy. Given the non-significant correlation between the joint SE and the empathic concern scale of the IRI, the reason why the effect showed a positive association with the EQ appears to be the presence of numerous items within the EQ designed to gage cognitive perspective taking. Theoretical accounts of empathy hold that it reflects feelings of concordance between self and other. According to Gallese (2001, p. 43), for example, empathy “is relevant when accounting for all aspects of behavior enabling us to establish a meaningful link between others and ourselves” and he further speculated that it is grounded in a human mirror neuron system that automatically simulates others’ emotional experience. While mindful of the need for caution in interpreting a null finding, our results could thus be taken to mean that the similarity effects driving the joint SE rely much more on the controlled and self-referential aspects of empathy (i.e., perspective taking and theory-of-mind) than the affective ones. A related suggestion would be that cognitive empathy contributes not just to the perception of similarity between acquainted co-actors but to the selection of event features that discriminate between them. Such a proposal is in line with evidence that the brain regions underpinning cognitive empathy also play a role in self-awareness (Decety and Sommerville, 2003). For example, neuroimaging studies have found that the theory-of-mind network becomes activated when people inhibit their automatic imitation of observed actions (e.g., Spengler et al., 2009) and strive to differentiate between their own actions and emotions and those of other people (e.g., Schulte-Rüther et al., 2007). A role of self/other differentiation in the joint SE was demonstrated by Dolk et al. (2011). Their study used an auditory version of the joint Simon task and aimed to manipulate the extent to which participants integrated the actions of their co-actor’s hand into their own body representation using either synchronous or asynchronous stroking. Results showed that the joint SE was greater following asynchronous stroking, in line with the idea that it reflects segregation of self- versus other-related information.

Notably, there was no significant influence of perspective taking on the SE when friends performed the task on their own despite the fact that the effect was not reliably smaller than in the earlier joint condition. The phenomenon of a SE in the absence of a co-actor mimics findings from previous research whereby the effect was observed even when the co-actors were unable to see or hear each other (e.g., Tsai et al., 2008; Vlainic et al., 2010). It could be argued that the individual SE reflected a carrying forward of spatial response codes acquired during the earlier joint paradigm, in line with evidence of similar transfer effects when participants proceed to a go/no-go version of the Simon task after previously having performed the standard two-handed version (Ansorge and Wühr, 2009; and see Sellaro et al., 2013, for a response-discrimination account of the Tsai et al., 2008 findings). However, the finding that the individual SE for friends was correlated positively with fantasizing raises the possibility that participants continued to conceptualize the task as a joint activity^1^. This being the case, it could be surmised that different processes underpinned friends’ performance in the joint- versus individual tasks; specifically, such that they considered their co-actor’s agency and intentions when he or she was physically present but treated them as a spatial referent (in their imagination) when they were not. Alternatively, whether the co-actor was present might have influenced the type of social attributes that friends emphasized in their event representations (with a shift from psychological features in the joint condition to physical features in the individual condition).

A reliable interaction between group and personal distress in relation to the individual SE reflected the fact that its magnitude was diminished for strangers with higher levels of personal distress. We are reluctant to place much weight on this finding given the failure of any of the empathy measures to predict the SE for strangers in the joint condition but we speculate that greater personal distress was linked with more self-focused attention. An association been distress and self-focused attention has been reported widely in the clinical psychology literature (e.g., Wells and Matthews, 2015) and may have been accentuated for strangers relative to friends if they felt less relaxed during the test session.

## Conclusion

In summary, we have reported a novel demonstration that the magnitude of the SE in the joint Simon task is predicted by cognitive perspective taking but *only* when the co-actors are friends. Given our relatively small sample size, the present findings must be regarded as preliminary. Nevertheless, we believe they highlight the importance of heeding the relationship between participants in studies of human interaction. If social similarity activates empathic processes then research using co-actors who are unfamiliar with one another might fail to capture the whole story regarding the nature of joint action. As we have discussed, our findings are amenable to interpretation in terms of both social (e.g., task co-representation) and non-social (e.g., referential coding) accounts. Even the latter explanation, though, still recognizes the important contribution of social factors to the joint SE by virtue of their role in shaping and discriminating between action event representations.

Future research could extend the present work in a number of ways. For example, the effects of friendship on the joint Simon task could be explored in more detail by gaging performance as a function of how long friends have known each other. Rather than simply comparing friends and strangers, this approach would reveal whether longer durations of friendship result in larger effects of perspective-taking skills on the joint SE. Likewise, varying the length of time before participants engage in the individual version of the task (i.e., following the joint task) would help to evaluate the suggestion that the SE for the individual condition reflects spatial response coding as (presumably) such an effect should tend to dissipate over time.

It would also be informative to compare the impact of perspective taking on the joint SE across different variants of the Simon task. Philipp and Prinz (2010) devised a paradigm with diminished spatial characteristics; here, participants were asked to respond to targets superimposed over task-irrelevant faces that corresponded to either their own face or the co-actor’s face. Testing pairs of friends, they demonstrated that participants reacted more slowly when the ‘go’ target appeared on the co-actor’s face, although such impairment disappeared when the task was performed individually. Based on the present findings, we anticipate that this effect will be positively correlated with perspective taking in the case of friends but not strangers. In contrast, we foresee *no* influence of perspective taking on the size of the joint SE in paradigms where the co-actor is an inanimate object.

## Conflict of Interest Statement

The authors declare that the research was conducted in the absence of any commercial or financial relationships that could be construed as a potential conflict of interest.

## Footnotes

1 We acknowledge the need for further research to confirm that fantasizing fails to predict the individual SE among friends when the individual condition *precedes* the joint condition.
